# Electrospun Multilayer Scaffolds Based on Poly (L-Lactic Acid) and Poly (Acrylonitrile) Reinforced with CaO Nanoparticles for Enhanced Skin Regeneration and Wound Healing

**DOI:** 10.3390/polym18080960

**Published:** 2026-04-15

**Authors:** Eugenio Rivera, Lissette Montoille, Fabián Guajardo, Fabian Álvarez-Carrasco, Sebastián Romero, Mauricio Gómez-Barrena, Esmeralda Lopez, Carlos Loyo, Claudio García-Herrera, Paula A. Zapata, Diana Zárate-Triviño, Juan José Martinez, Daniel A. Canales

**Affiliations:** 1 Laboratorio de Biomecánica y Biomateriales, Departamento de Ingeniería Mecánica, Facultad de Ingeniería, Universidad de Santiago de Chile, Santiago 9170022, Chilefabian.alvarez.c@usach.cl (F.Á.-C.); 2 Instituto de Ciencias Naturales, Facultad de Medicina Veterinaria y Agronomía, Universidad de Las Américas, Manuel Montt 948, Santiago 7500975, Chile; 3 Grupo Polímeros, Departamento de Ciencias del Ambiente, Facultad de Química y Biología, Universidad de Santiago de Chile (USACH), Casilla 40, Correo 33, Santiago 9170022, Chile; esmeralda.lopez@usach.cl (E.L.);; 4 Centro de Investigación y Desarrollo en Ciencias de la Ingeniería (CIDCI), Escuela de Ingeniería Civil y Ciencias Geoespaciales, Facultad de Ingeniería Ciencia y Tecnología, Universidad Bernardo O’Higgins, Av. Viel 1497, Santiago 8370993, Chile; mauricio.gomez.b@usach.cl; 5 Departamento de Ingeniería Geoespacial y ambiental, Facultad de Ingeniería, Universidad de Santiago de Chile, Santiago 9170022, Chile; 6 School of Chemical Sciences and Engineering, Yachay Tech University, Hda. San José s/n y Proyecto Yachay, Urcuquí 100119, Ecuador; 7 Facultad de Ingeniería y Ciencias, Universidad Adolfo Ibañez UAI, Diagonal Las Torres 2640, Santiago 7941169, Chile; 8 Laboratorio de Inmunología y Virología, Facultad de Ciencias Biológicas, Universidad Autónoma de Nuevo León, San Nicolás de los Garza 66455, Mexico; 9 Centro de Ingeniería y Desarrollo Industrial, Av. Playa Pie de la Cuesta No.702, Desarrollo San Pablo, Santiago de Querétaro 76125, Mexico; 10 Centro de Investigación en Ciencias Biológicas y Químicas, Universidad de Las Américas, Manuel Montt 948, Santiago 7500975, Chile

**Keywords:** electrospun matrices, multilayer architectures, PLA/PAN blends, CaO nanoparticles, skin tissue regeneration

## Abstract

This study reports the development and characterization of hierarchical electrospun scaffolds based on poly (L-lactic acid) (PLA) and polyacrylonitrile (PAN) reinforced with calcium oxide (CaO) nanoparticles (18.5 ± 4.7 nm) for skin regeneration. Six configurations, including two five-layer multilayer systems (PLA/PAN/CaO and PAN/PLA/CaO), were evaluated to determine how composition and deposition sequence influence physicochemical, mechanical, and biological performance. FT-IR, XRD and DSC confirmed the successful integration of CaO, while thermal analysis evidenced an effect of chain mobility and interfacial interactions within multilayer systems. Cross-sectional SEM revealed the presence of both fibers with continuous interfaces. Nitrogen adsorption showed that CaO significantly increased the specific surface area (e.g., from 4.6 m^2^/g in neat PLA to 21.65 m^2^/g in PLA/CaO), with type IV isotherms indicating mesoporosity. Wettability assays demonstrated reduced contact angle in PLA (from 126.3° to 91.8°) and sequence-dependent surface properties in multilayers. Tensile testing confirmed that the multilayer architecture bridged the mechanical gap between compliant PLA and high-strength PAN, yielding intermediate moduli (~10–11 MPa) and balanced toughness. Antibacterial assays against *S. aureus* and *E. coli* showed that CaO significantly reduced bacterial viability, with PLA/PAN/CaO achieving the highest inhibition (up to 37.1%). In vitro HaCaT assays and in vivo implantation in BALB/c mice confirmed high cytocompatibility and biocompatibility. These findings demonstrate that multilayer electrospinning of PLA/PAN/CaO enables the design of structurally integrated, bioactive, and mechanically balanced scaffolds for advanced wound healing and dermal repair.

## 1. Introduction

The skin is the body’s largest organ and functions as a multifunctional barrier that protects underlying tissues from pathogens, chemical exposure, and mechanical trauma while regulating hydration and thermoregulation [1,2]. Although skin exhibits an intrinsic capacity for repair, extensive injury and chronic systemic conditions (e.g., diabetes and vascular disease) can disrupt the normal healing cascade, leading to acute wounds with delayed closure or to chronic, non-healing ulcers that represent a major clinical and socioeconomic burden, with annual treatment costs that can exceed $1 billion in some health-care systems [3,4]. Conventional management, including passive dressings, autografts, allografts/xenografts, and commercial skin substitutes (e.g., Integra^®^, Apligraf^®^), often fails to provide the combined requirements of mechanical protection, moisture balance, infection control, and pro-regenerative bioactive cues needed for coordinated re-epithelialization and dermal regeneration [5]. In particular, autologous grafting is limited by donor-site morbidity and scarring, whereas allogeneic and xenogeneic grafts can be constrained by immunogenicity and disease-transmission concerns; additionally, many commercial products remain costly and may require multiple procedures, motivating the development of advanced biomaterial-based strategies for wound healing [3,6].

These limitations have fueled interest in tissue-engineered scaffolds that recapitulate key features of the extracellular matrix (ECM) to support coordinated skin regeneration [7]. Tissue engineering (TE) addresses wound-healing challenges by designing biomaterials that combine structural support with biological cues, thereby creating a favorable microenvironment for repair. In wound management, an ideal TE-based dressing or scaffold should provide physical protection and moisture balance while promoting endogenous regenerative processes (such as angiogenesis, cell proliferation, and re-epithelialization) and simultaneously mitigating adverse factors including microbial contamination and excessive inflammation [8]. This rationale has driven the development of bioactive dressings and three-dimensional scaffolds intended to overcome the shortcomings of standard treatments [9]. Among the manufacturing technologies explored for this purpose, electrospinning has attracted considerable attention due to its versatility and biomimetic potential, enabling the production of continuous fibrous mats with fiber diameters ranging from the nanometer to micrometer scale [1,10]. Electrospun nanofibers typically exhibit high porosity and interconnected pore networks, yielding a high surface-to-volume ratio that supports gas exchange and exudate management (key requirements for wound dressings) while providing physical barrier microorganisms [11,12]. Importantly, this fibrous architecture resembles the native ECM and can enhance cell adhesion, migration, and proliferation, providing a biophysical niche to tissue regeneration and accelerated wound closure [5]. Beyond structural mimicry, electrospun scaffolds can act as delivery platforms for therapeutic agents, including antimicrobial compounds, anti-inflammatory molecules, and growth factors, enabling multifunctional wound dressings that combine protection with active biological modulation [9].

Electrospun fibers derived from natural polymers (e.g., collagen and chitosan) as well as synthetic materials such as poly (lactic acid) (PLA) and polycaprolactone (PCL) have been widely investigated as scaffolds due to their ECM-like fibrous architecture and processing versatility [9,12]. Among these, PLA is a thermoplastic aliphatic polyester extensively used in TE because of its biocompatibility and hydrolytic biodegradation, enabling gradual resorption in vivo [13]. PLA can provide sufficient mechanical integrity for handling and temporary structural support and has a long history of regulatory acceptance in biomedical devices. Prior studies have reported that electrospun PLA-based mats are promising substrates for skin repair and wound healing applications [14,15]. However, electrospun PLA often exhibits limited cell–material interactions due to its relatively hydrophobic surface, and its stiffness may not fully match the compliant mechanical behavior of native dermal tissue. To address these constraints, PLA is commonly combined with other polymers via approaches such as blending, copolymerization, or layered architectures to tailor wettability and mechanical response [16,17]. In this context, polyacrylonitrile (PAN) has attracted interest as a mechanically robust and chemically stable polymer that can be readily processed into uniform electrospun fibers using polar aprotic solvents such as N,N-dimethylformamide (DMF) or dimethyl sulfoxide (DMSO) [18,19]. Accordingly, combining PAN with PLA to overcome PLA’s limitations by improving functional performance (e.g., surface properties and mechanical stability) in biofunctional scaffolds and controlled-release platforms for TE [17].

Electrospinning further enables the fabrication of multilayer (ML) scaffolds, where immiscible materials can be deposited sequentially to generate stratified constructs with controlled composition across thickness. By adjusting the number of layers, individual layer thickness, and deposition sequence, ML architecture can be used to modulate mechanical behavior and biological performance in a design-dependent manner. At the same time, they can provide a practical route to physically integrate components that are difficult to blend homogeneously in a single solution and retain the distinct functions of each layer [20]. Prior studies illustrate the versatility of this approach: Shao et al. employed a multilayer design to combine electrospun PLA with Tussah silk fibroin, achieving improved mechanical performance in comparison to the corresponding single-polymer mats. The design was able to maintain cytocompatibility, highlighting the ability of layer-by-layer deposition to tune scaffold mechanics even for solution-immiscible systems [21]. More directly relevant to wound care, Khan et al. reported an ML electrospun dressing integrating functionalized PAN as a structural barrier layer, TiO_2_ nanoparticles as an antibacterial component, and gelatin as a biocompatible contact layer, resulted in favorable porosity and wettability, high fibroblast viability, and broad antimicrobial activity against multiple pathogens [22]. Collectively, these studies support multilayer electrospinning as a robust strategy to integrate complementary materials and tailor scaffold performance for biomedical applications, including wound healing [23,24].

Beyond polymer selection and scaffold architecture, the incorporation of bioactive inorganic nanoparticles is widely used to tailor surface properties and provide biochemical cues that support cell adhesion, proliferation, and tissue regeneration [12]. In skin repair, calcium signaling coordinates key events including fibroblast activity, keratinocyte migration, and angiogenesis, thereby influencing re-epithelialization and extracellular matrix remodeling. Accordingly, calcium-based materials have been explored in wound dressings as local sources of Ca^2+^ with potential to promote collagen deposition and tissue contraction under wound-relevant conditions [25,26]. Calcium oxide (CaO) nanoparticles have therefore been explored as multifunctional additives in electrospun polymeric matrices, where they can modify physicochemical properties and confer antimicrobial or pro-regenerative effects depending on concentration and dispersion [27,28,29]. For instance, Canales et al. reported electrospun PLA/CaO nanocomposites (10–20 wt%) forming porous fibrous mats with preserved structural integrity, alongside improved biological responses and measurable antibacterial activity against common pathogens [30]. More directly relevant to skin regeneration, Varela et al. developed electrospun PAN/CaO composites derived from scallop-shell CaO (5–20 wt%) and reported cytocompatibility, enhanced cell-related outcomes (including viability and migration), and improved fibrovascular tissue formation in vivo, supporting the translational potential of CaO-containing electrospun scaffolds for soft-tissue repair [31].

In this study, we combine these concepts by fabricating electrospun scaffolds based on PLA and PAN as single-polymer mats and as CaO-containing systems, including multilayer architectures, to evaluate how composition and deposition sequence influence physicochemical, mechanical, and biological performance relevant to wound healing. Specifically, six formulations were investigated: PLA, PAN, PLA/CaO, PAN/CaO, PLA/PAN/CaO, and PAN/PLA/CaO.

## 2. Materials and Methods

### 2.1. Materials

Poly (L-lactic acid) (PLLA) filament (Sakata 3D brand, Granada, Spain) containing less than 5% of the D-isomer, as determined by proton nuclear magnetic resonance spectroscopy (^1^H-NMR), was used. The polymer exhibits an average molecular weight (Mw) of 335,173 g/mol, and in the manuscript it is described as a PLA. Polyacrylonitrile (PAN) with an average molecular weight of 150,000 g/mol was purchased from Sigma-Aldrich (St. Louis, MO, USA). Calcium oxide (CaO) nanoparticles were previously obtained from *Argopecten purpuratus* clamshell waste and characterized by SEM, FT-IR and DRX, exhibiting a spherical morphology and an average size of 18.5 ± 4.7 nm [31].

### 2.2. Preparation of PLA, PAN, PLA/CaO and PAN/CaO Solutions

For PLA, a 10% (*w*/*v*) solution was obtained by dissolving the polymer in a 9:1 (*v*/*v*) mixture of chloroform (CF, Winkler Ltd., 99.8% purity, Santiago, Chile) and N,N-dimethylformamide (DMF, Merck, 99.9% purity, Darmstadt, Germany). For PAN, a 6% (*w*/*v*) solution was prepared using DMF as the solvent. CaO nanoparticles were incorporated at a fixed concentration of 10% *w*/*w* relative to the weight of PLA and PAN. Initially, the required amount of CaO was dispersed in 5 mL of the respective solvents and subjected to ultrasonic treatment for 1 h. Subsequently, the corresponding amount of each polymer was added, and the total solvent volume was adjusted for each solution. The mixture was continuously stirred for 24 h to ensure complete dissolution and homogeneity.

### 2.3. Preparation of PLA, PAN, PLA/CaO, PAN/CaO and Hybrid PLA/PAN/CaO and PAN/PLA/CaO Matrices by Electrospinning

The electrospinning process was carried out using a TL-01 electrospinning machine (TongLi-Tech, Changzhou, China) with an applied voltage of 20 kV. The flow rate of the PLA and PLA/CaO solutions was set at 2 mL/h, while that of the PAN and PAN/CaO solutions was 1.6 mL/h. The polymer solutions were loaded into 20 mL syringes fitted with 22-gauge needles, maintaining a fixed distance of 15 cm between the needle and the manifold. All experiments were performed at room temperature (22–25 °C, 40–50% relative humidity).

The PLA/PAN/CaO and PAN/PLA/CaO hybrid matrices were produced using a multilayer deposition design with identical preparation and electrospinning conditions for each polymer. Two multilayer configurations were developed, each composed of five layers: (1) PLA/CaO-PAN/CaO-PLA/CaO-PAN/CaO-PLA/CaO, hereafter referred to as PLA/PAN/CaO, and (2) PAN/CaO-PLA/CaO-PAN/CaO-PLA/CaO-PAN/CaO, hereafter referred to as PAN/PLA/CaO. The electrospinning time was adjusted to ensure an equivalent polymer mass per layer. Specifically, the PLA layers were electrospun for 60 min and the PAN layers for 125 min, yielding approximately 200 mg of polymer per layer. The electrospun fibers were collected on parchment paper, labeled, and kept refrigerated until further use.

### 2.4. Structural and Physicochemical Characterization

To obtain representative measurements of the structural and physicochemical properties of the fibers, a minimum of three samples was established for sampling and analysis. In specific cases, this number was increased to five samples to enhance statistical reliability.

#### 2.4.1. Morphology and Fiber Size

A field emission scanning electron microscope (FE-SEM, Gemini SEM360, Carl Zeiss AG, Oberkochen, Germany) was employed to analyze the surface morphology of the samples. Cross-sectional images were performed by Zeiss SEM, model EVO I MA10 (Carl Zeiss AG, Oberkochen, Germany). Prior to imaging, the samples were sputter-coated with a conductive layer of approximately 5 nm thickness to enhance electrical conductivity.

Fiber diameters were measured using ImageJ software (v 1.48q, National Institutes of Health, Bethesda, MD, USA), based on a minimum of 200 individual measurements taken at randomly selected positions along the fibers. A total of three samples were analyzed in this study.

#### 2.4.2. Infrared Spectroscopy (FT-IR) and X-Ray Diffraction (XRD)

The chemical composition of the electrospun fibers was analyzed via Fourier Transform Infrared Spectroscopy (FT-IR) (Perkin Elmer, Spectrum Two, Waltham, MA, USA) to obtain a typical peak associated with PLA, PAN and CaO. This analysis was used as a control for all fiber samples obtained (n ≥ 3). The measurements were conducted over a wavenumber rage 400–4000 cm^−1^, using air as the background at room temperature, with a resolution of 1 cm^−1^ and 30 scans average.

In addition, X-ray diffraction (XRD) patterns data were obtained on a Miniflex600 diffractometer (Rigaku Corporation, Tokyo, Japan), equipped with a D/tex Ultra2 detector; the X-ray generator was operated at 40 kV and 15 mA, using a sealed tube CuK*α* radiation source.

#### 2.4.3. Porosity and Surface Area

For porosity analysis, three samples were evaluated for each measurement. Prior to analysis, the sample were degassed at 323 K for 8 h. A Micromeritics 3Flex (v 4.02, Norcross, GA, USA) was employed to perform the nitrogen (N_2_) adsorption–desorption isotherms at 77 K. Total pore volume was determined within the relative pressure range of P/P_0_ = 0–0.99, while the average pore size was calculated in the range of P/P_0_= 0–0.3 using the Barrett–Joyner–Halenda (BJH) model. The Brunauer–Emmett–Teller (BET) method was applied to estimate the apparent surface area.

#### 2.4.4. Wettability

Wettability and water uptake were evaluated using a tensiometer (ThetaLite Attention, Biolin Scientific AB, Hängpilsgatan 7, SE-426 77 Västra Frölunda, Sweden) to measure the static contact angle. For the analysis, a 4 μL drop of distilled water was placed on the surface of the matrices, the contact angle was determined using the Young la place equation after 5 samples.

#### 2.4.5. Thermal Analysis

Thermal properties were evaluated via thermogravimetric analysis (TGA) using a TGA 2 STARE System (Mettler–Toledo AG, Greifensee, Switzerland) equipment. At least three samples were heated from 25 °C to 800 °C at a rate of 10 °C/min under N_2_ atmosphere. Differential scanning calorimetry (DSC) was performed on a DSC 1 STAR+ System (Mettler–Toledo AG, Greifensee, Switzerland) from 0 °C to 400 °C at 10 °C/min. Only the heating scan was analyzed, as it reflects the inherent crystallinity of the electrospun fibers, while the second scan would erase the thermal history associated with the electrospinning process. The glass transition temperature (T_g_), crystallization temperature (T_c_), and melting temperature (T_m_) were measured, and the degree of crystallinity grade (X_c_) was calculated, using the following formula:

$$X_{c}\left(\%\right)=\frac{\Delta H_{m}-\Delta H_{cc}}{\Delta H{{}^{\circ}}_{m}}\times \frac{1}{W}\times 100$$

where W is mass fraction of polymer.

Since PLA was the only polymer to exhibit crystallinity, its degree of crystallinity (X_c_) was calculated based on the theoretical melting enthalpy (ΔH°_m_) of 93.6 J g^−1^ for 100% crystalline PLA [32].

#### 2.4.6. Mechanical Characterization by Uniaxial Tensile Testing

The tensile response of the electrospun mats was assessed on a BioTester 5000 biaxial testing machine (CellScale, CellScale Biomaterials Testing, Waterloo, ON, Canada) equipped with a 10 N load cell, following the ASTM D882-12 standard for thin films (thickness < 1 mm) [33]. For each of the six formulations, five rectangular specimens were randomly excised from different regions of the electrospun mats to account for potential spatial variability. During testing, samples were gripped at their ends and pulled at a constant crosshead speed of 0.5 mm/min. The tests were not extended until complete rupture; instead, each test was terminated when the measured force decreased by 10% from Ultimate Tensile Strength (UTS). Force and displacement data were converted to engineering stress (σ) and strain (ε). The elastic modulus was obtained from the slope of the linear elastic region by linear regression. Ultimate tensile strength (UTS) was taken as the maximum stress reached, and the deformation at UTS (εUTS) and toughness were calculated from the area under stress–strain curves up to UTS. All measurements were performed at room temperature.

### 2.5. In Vitro Biological Characterization

#### 2.5.1. Relative Bacterial Viability

The antibacterial activity of electrospun fiber based on PLA, PAN, PLA/CaO, PAN/CaO and hybrid multilayer PLA/PAN/CaO and PAN/PLA/CaO was determined against *S. aureus* (Gram-positive) and *E. coli* (Gram-negative) bacteria. The bacteria strains were incubated in medium at 37 °C during 24 h, and an optical density (OD) of bacteria of 0.015 according to turbidity measurements of bacterial cultures using a UV equipment at 600 nm (Thermo Scientific TM GENESYS 30TM, Thermo Fisher Scientific, Waltham, MA, USA). The fiber mats were cut into a square of 10 mm × 10 mm (20 mg of sample) and sterilized by UV radiation for 1 h. The samples were placed into a falcon tube of 10 mL with 2 mL of bacteria suspension and incubated at 37 °C for various times (3 and 6 h). The OD values were determined by taking a 1 mL of bacteria suspension after each time interval and measured at 600 nm, and the relative bacterial viability was calculated using the following equation:

$$Relative\;bacterial\;viability\left(\%\right)=\frac{O.D.Sample}{O.D.Control}\times 100$$

#### 2.5.2. Cell Viability

The cytocompatibility of the scaffolds was assessed using human keratinocyte (HaCaT) cell lines cultured in DMEM (Thermo Fisher Scientific, Waltham, MA, USA) supplemented with 10% *v*/*v* fetal bovine serum (Thermo Fisher Scientific, Waltham, MA, USA) and 1% *v*/*v* penicillin–streptomycin (Thermo Fisher Scientific, Waltham, MA, USA). Cells were incubated at 37 °C in a humidified atmosphere containing 5% CO_2_ for 24 h and used at 60–70% confluence. Prior to seeding, scaffold samples (15 × 15 mm) were mounted on custom-designed cell holders fabricated via 3D printing using PLLA filament. These holders were sterilized by immersion in 70% ethanol for 24 h, followed by UV exposure for 1 h on each side.

Scaffold was placed in a well of a 24-well plate, and 45,000 cells were seeded per holder. The cultures were incubated at 37 °C for 24, 48, and 42 h. Cell viability was assessed using the MTT assay, employing the reagent 3-(4,5-dimethylthiazol-2-yl)-2,5-diphenyltetrazolium bromide (Merck KGaA, Darmstadt, Germany). The reagent was diluted 1:10 in supplemented DMEM, and 400 μL of this solution was added to each well. After 3 h of incubation in the dark at 37 °C, the medium was removed, and 400 μL of DMSO (Supelco, Merck KGaA, Darmstadt, Germany) was added to dissolve the formazan crystals (Sigma-Aldrich, St. Louis, MO, USA). The solution was then agitated for 6 min and transferred to a 96-well plate for absorbance measurement at 570 nm using a microplate reader (Infinite 150, Tecan Group Ltd., Männedorf, Switzerland).

#### 2.5.3. Cell Migration

For cell migration assay, Human Keratinocyte (HaCaT) cells were seeded in a 12-well plate and incubated at 37 °C with 5% CO_2_. Once the cells reached confluence, a scratch was created using a 200 μL pipette tip, and cell debris was thoroughly washed away with PBS. HaCaT Cells used as control were fed with only media, while other groups were exposed to electrospun matrices, placed in transwell inserts. The scratch defects were imaged to monitor cell migration using an inverted microscope at different time points (0 h, 3 h, 6 h, 12 h, 24 h, 48 h, and 72 h). The images were analyzed using ImageJ software (v 1.48q, National Institutes of Health, Bethesda, MD, USA) to estimate the area of migrated cells.

### 2.6. In Vivo Biological Characterization

#### Biocompatibility Analysis

To evaluate the in vivo biocompatibility of the electrospun fibers, one-month-old BALB/c mice weighing approximately 30 g were used as the animal model. A subdermal implantation approach was employed. For each scaffold type, three mice were randomly selected. Subcutaneous incisions measuring 1 cm in length were made, and subdermal pockets approximately 1 cm long and 3 cm deep were created. The scaffolds were implanted into these pockets and left in place for 14 days. After the implantation period, the samples were retrieved and fixed in buffered formalin for 48 h. They were then rinsed with phosphate-buffered saline (PBS) through three 10 min washes before being processed for histological sectioning and analysis.

### 2.7. Statistical Analysis

Values are expressed as mean ± SD. Statistical analyses were performed using GraphPad Prism 5.0 software (GraphPad Software, San Diego, CA, USA). Normality was assessed using the Shapiro–Wilk test. Homogeneity of variances was evaluated using Bartlett’s test when data met normality, or Levene’s test when normality was not met. A *p*-value > 0.05 was considered indicative of normality and/or homoscedasticity for each dataset. For data meeting parametric assumptions, differences among groups were analyzed using one-way ANOVA followed by Bonferroni’s multiple-comparison post hoc test (all pairwise comparisons). For datasets violating parametric assumptions, the nonparametric Kruskal–Wallis test was applied, followed by Dunn’s post hoc test for multiple comparisons. Statistical significance was defined as *p* < 0.05 (*), *p* < 0.01 (**) or *p* < 0.001 (***).

## 3. Results

### 3.1. Morphology and Fiber Size

**Figure 1:**
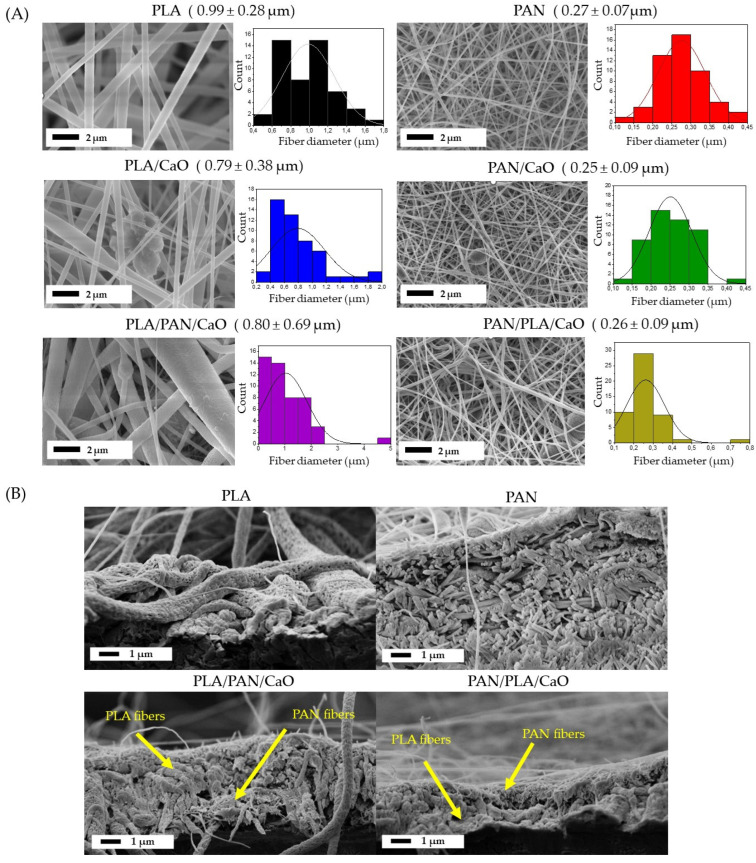
SEM Images of electrospun matrices (**A**) Surface morphology and fiber-diameter histograms and (**B**) Cross-sectional morphology only for PLA, PAN, PLA/PAN/CaO and PAN/PLA/CaO.

**Figure 2:**
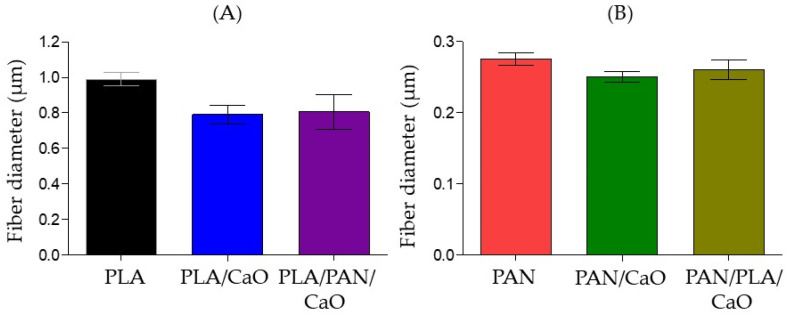
Analysis of fiber diameter of (**A**) PLA based fibers; (**B**) PAN based fibers (n = 100).

The morphological analysis of the electrospun fibers was performed using Scanning Electron Microscopy (SEM) and is summarized in Figure 1. For the pure PLA and PAN matrices, homogeneous, uniform fibers without defects were observed in both cases, forming a porous network with interconnected pores. A greater difference in size distribution was observed between the two matrices, reaching average fiber diameters of 0.99 ± 0.28 µm and 0.28 ± 0.07 µm (Figure 2), respectively, values very similar to those previously reported. This marked difference in size is related to the nature of the solution and the physicochemical properties of each polymer, where the higher conductivity of PAN and the volatility of the solvent used (DMF) promote more efficient electrostatic stretching, resulting in thinner fibers [34]. Analysis of PLA/CaO and PAN/CaO composite systems reveals that the incorporation of CaO nanoparticles introduces morphological alterations in the form of aggregates and surface defects in the fibers. These irregularities are primarily attributed to the agglomeration of the nanoparticles within the polymer solution before or during the electrospinning process, disrupting the uniformity of the fibrous surface observed in the pure polymer samples. This agglomeration is related to the low nanoparticle-matrix interaction, as previously reported [30,31].

For multilayer hybrid matrices (PLA/PAN/CaO and PAN/PLA/CaO), SEM micrographs only allow visualization and measurement of the outermost layer. Consequently, surface analysis fails to discern a bimodal diameter distribution, as it only captures the morphology of the polymer deposited on the surface [17].

To validate the multilayer architecture of the electrospun scaffolds, cross-sectional SEM micrographs were performed (Figure 1B). This approach allowed for direct observation of the internal organization of the layers and complemented the surface analysis previously presented in Figure 1A.

The micrographs reveal a stratified structure, where it is possible to distinguish regions with fibrous morphologies characteristic of each polymer. In particular, the PLA layers exhibit larger diameter fibers, consistent with the previously obtained diameter distribution results (~0.8–1.0 µm), along with evident surface porosity.

On the other hand, the PAN regions exhibit a network of significantly thinner fibers (~0.25–0.30 µm), homogeneous and with smooth surfaces, showing no evidence of surface porosity. These morphological differences between the two layers allow for clear identification of each component within the multilayer assembly.

For hybrid scaffolds (PLA/PAN/CaO and PAN/PLA/CaO), a continuous interface is observed between adjacent layers, without evident discontinuities, suggesting adequate interfacial adhesion between the polymers. This behavior may be associated with sequential deposition during the electrospinning process, which promotes partial interpenetration of fibers at the interfaces, improving the structural cohesion of the system [35].

### 3.2. Infrared Spectroscopy and X-Ray Diffraction

**Figure 3:**
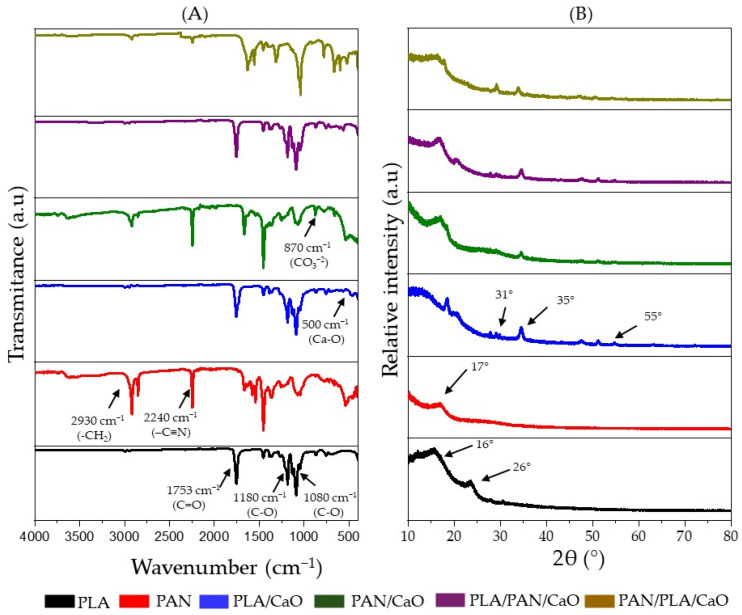
Physicochemical characterization of electrospun fibers, (**A**) Infrared spectroscopy (IR); (**B**) X-Ray Diffraction analysis (XRD).

Chemical analysis using FT-IR spectroscopy was performed to confirm the characteristic signals of the constituent polymers and the successful incorporation of CaO nanoparticles into the matrices, as shown in Figure 3A. The spectrum of pure PLA reveals the characteristic absorption bands associated with its ester structure. The most intense signal, consistently located at 1752 cm^−1^, is attributable to the carbonyl bond (C=O) stretching vibration. Meanwhile, the ester (C-O) bond stretching is evident in the 1180–1080 cm^−1^ range. Other key absorptions include the C-H bond stretching vibration of the methyl group at 1453 cm^−1^ and a signal at 1360 cm^−1^ related to C-C bond stretching [32,36]. In the case of PAN, its distinctive bands are clearly identifiable. The symmetric stretching vibration of the methylene group CH_2_ is located at 2930 cm^−1^; the absorption crucial for the identification of the polymer, corresponding to the stretching vibration of the nitrile group (-C≡N), appears at 2240 cm^−1^ [31,37]. The incorporation of CaO nanoparticles is evident in the spectra of all nanocomposite matrices with the base polymers and the hybrid matrices with a multilayer structure. Prominent peaks are observed at 1413 cm^−1^ and 874 cm^−1^, which are assigned to the asymmetric vibration of the carbonate group (CO_3_^2−^) (calcite). Additionally, a signal at 510 cm^−1^ is indicative of the Ca-O bond vibration. These new absorptions confirm the presence of the calcium compound in the electrospun matrices [38].

On the other hand, XRD analysis was used to characterize the crystalline state of the polymers and corroborate the crystalline nature of the incorporated CaO nanoparticles as shown in Figure 3B. The diffraction patterns of the pure polymers (PLA and PAN) exhibit the typical morphological characteristics of electrospinning. PLA shows a broad, diffuse reflection centered around 16° (2θ), suggesting a predominantly amorphous or semicrystalline structure with low crystallinity. PAN, on the other hand, shows a sharp reflection near 17° (2θ), associated with its crystalline phase [39,40]. The incorporation of CaO nanoparticles into the composite fibers is confirmed by the appearance of multiple sharp signals in the diffraction pattern, which are characteristic of inorganic crystalline phases. Specifically, notable peaks were observed at 31, 35, and 55° (2θ). These signals correspond to the crystallographic planes (100), (101), and (110), respectively [27,30].

### 3.3. Porosity and Surface Area

**Figure 4:**
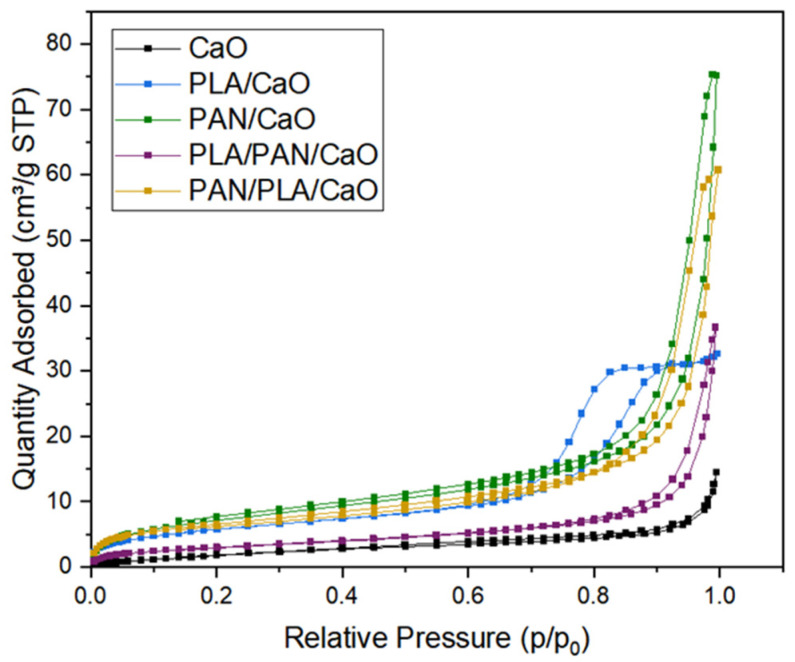
Porosity N_2_ isotherm curves of CaO nanoparticles and electrospun fibers of PLA/CaO, PAN/CaO, PLA/PAN/CaO, and PAN/PLA/CaO.

Electrospun porous fibers hold significant clinical potential, particularly for the management of complex wounds such as chronic ulcers, burns, and diabetic foot lesions. Their interconnected porosity enables efficient gas exchange and adsorption of wound exudate, creating a favorable environment for cell proliferation and angiogenesis. Additionally, the porous structure facilitates cell growth and gas exchange while acting as an effective barrier against external microorganisms, thereby helping to protect the wound from infection [41]. Figure 4 shows the N_2_ adsorption isotherm at 77 K for CaO and the hybrid electrospun fibers composed of PLA, PAN and CaO. Most samples exhibit type IV isotherms with H3-type hysteresis, indicating a slit-like pore structure. This suggests that the electrospun fiber layers are relatively compacted. In contrast, the PLA/CaO sample exhibits a Type IV isotherm with an H1-type hysteresis loop, which is characteristic of a more uniform mesoporous material [17]. As has been seen in SEM analysis (Figure 1) macropores are observed, a feature feasible to observe in electrospun fibers, where the isotherm data implies that the fibers are relatively dense, with porosity limited primarily to the slit-like pores. The multilayer system PLA/PAN/CaO exhibited the lowest surface area, suggesting it is the most compact of the samples.

According to IUPAC [42], H3 hysteresis is associated with non-rigid aggregates of plate-like particles, which in electrospun mats can be interpreted as compacted layers with low internal porosity. Conversely, H1 hysteresis indicates a narrow distribution of uniform mesopores, making PLA/CaO the most structurally uniform sample regardless of its specific surface area.

As shown in Table 1, the specific surface area increased in the following order: CaO < PLA/PAN/CaO < PLA/CaO < PAN/PLA/CaO < PAN/CaO. The incorporation of Ca significantly altered the surface area. For instance, while neat PLA typically exhibits a low surface area (e.g., 4.6 m^2^/g) in our previous work, the addition of CaO increased this value to 21.65 m^2^/g. These variations are directly attributed to the specific interactions between each polymer and the CaO nanoparticles, as well as the interfacial dynamics within the multilayer array.

### 3.4. Wettability Analysis

**Figure 5:**
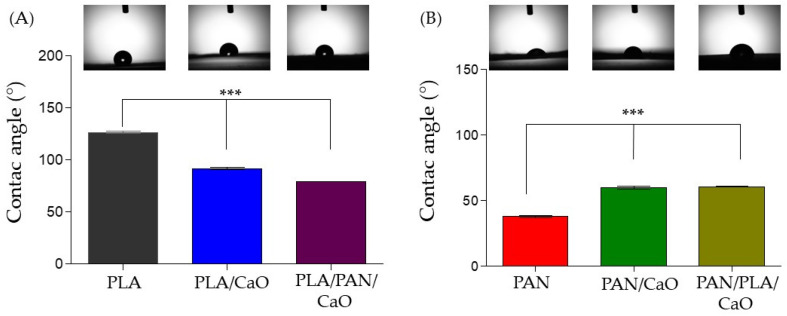
Wettability analysis of electrospun fibers by contact angle measurements (**A**) PLA based fibers; (**B**) PAN based fibers (n = 30), (*p* < 0.001, ***).

The interaction capacity between a biomaterial’s surface and the aqueous environment is a critical factor determining its potential for tissue regeneration. This interaction is evaluated by measuring the contact angle to define the hydrophilic or hydrophobic nature of the matrices (Figure 5). The PLA-based matrix exhibited marked hydrophobicity with a contact angle of 126.3°, a behavior associated with the low polarity of its carbon chains. This characteristic is often amplified in electrospun structures due to porosity and surface roughness, which hinder liquid penetration [43,44]. However, this response was significantly modified with the incorporation of CaO nanoparticles into the PLA/CaO system, where the angle decreased to 91.8°. This transition to a more wettable state is explained by the intrinsic hydrophilicity and high surface energy of the calcium oxide nanoparticles, which partially compensate for the nonpolar nature of the polymer matrix [30].

Conversely, the pure PAN-based matrix exhibited a strongly hydrophilic nature with a value of 37.7°, derived from the high polarity of the nitrile groups present in its structure [45]. When evaluating the reinforced PAN/CaO system, an increase in the angle to 59.8° was observed. This suggests that the presence of CaO alters the fiber topography in such a way that it slightly Inhibits droplet spreading compared to the smooth PAN fiber, while still maintaining the hydrophilic character of the material [31].

In multilayer systems, wetting behavior is primarily governed by the nature of the outer surface. In the PLA/PAN/CaO system, the 79.1° value reflects a balance between the hydrophobic barrier of the outer PLA layer and the influence of the internal hydrophilic layers loaded with CaO, resulting in a surface significantly more receptive to water than pure PLA. A similar surface dominance phenomenon is observed in the PAN/PLA/CaO system, whose 60.6° angle is almost identical to that of the binary PAN/CaO system, confirming that the final arrangement of the reinforced polyacrylonitrile layer is the determining factor in the immediate wettability response [17]. Taking together, these results demonstrate that combining multilayer deposition and CaO doping allows for modification of the surface energy compared to pure systems.

### 3.5. Thermal Analysis

**Figure 6:**
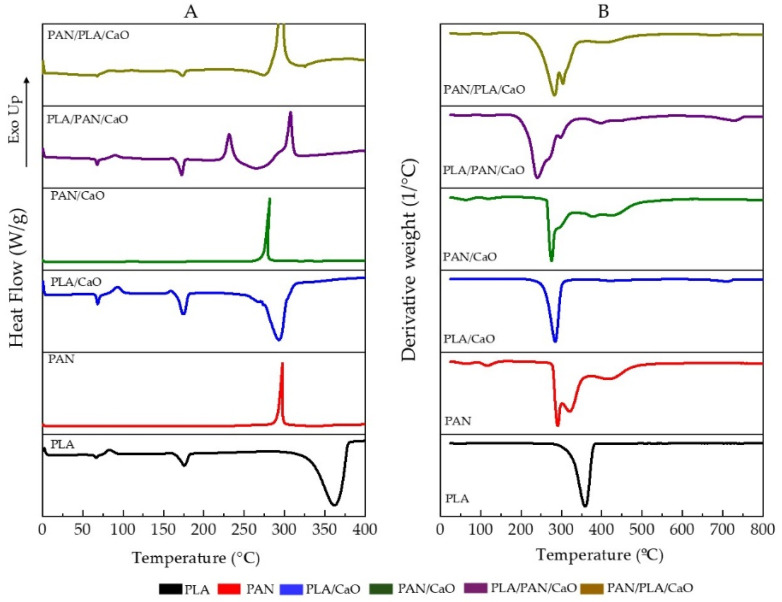
Thermal analysis of electrospun matrices (**A**) DSC thermograms range 0–400 °C and (**B**) TGA thermograms range 0–800 °C.

The DSC thermal analysis, summarized in Figure 6A, shows the values in Table 2, where the distinctive transitions associated with each component and their hierarchical integration can be found. Pure PLA exhibits typical thermal behavior (T_g_ ≈ 66.6 °C, T_cc_ ≈ 82.5 °C, T_m_ ≈ 175.6 °C), and a broad signal between 320 and 360 °C is observed, which can be attributed to the thermal decomposition of PLA [30]. PAN, on the other hand, does not show clearly detectable T_g_ under the selected conditions, but it does exhibit an exothermic transition at 295 °C related to the cyclization of the PAN structure, similar to what has been previously reported [46].

The incorporation of CaO into PLA increases both T_g_ and T_cc_, indicating a restricted chain mobility al low temperatures and a possible nucleating effect of the nanoparticles, while the slight decrease in T_m_ suggests that at high temperatures, nanoparticles can generate areas of greater free volume that facilitate the molecular disorganization of the chains, and the presence of CaO accelerates the PLA decomposition process [47]. For PAN/CaO, a decrease in the exothermic signal is also evident, indicating that CaO also modulates the thermal stability of the PAN phase, accelerating the cyclization process [48].

In multilayer systems, thermal responses depend on the layer configuration. For PLA/PAN/CaO, the effect of CaO was observed, increasing the T_g_ and T_cc_ values. A decrease in the PLA decomposition process was also observed, and the appearance of two exothermic signals associated with PAN was noted. This could indicate that the presence of PLA, and particularly the –COOH groups, could accelerate the cyclization process at the PLA/PAN interfaces [48]. For PAN/PLA/CaO, the same phenomenon is not observed. The PLA signals appear more diminished, which could be attributed to the lower amount of PLA, and only one exothermic signal is observed, although over a wider temperature range, increasing the ∆T (T_f_−T_i_). This suggests the interfacial interaction between PAN/PLA and the presence of CaO modifies the cyclization process. For multilayer architecture, the modification on thermal transitions depending on the stacking sequence (PLA/PAN/CaO or PAN/PLA/CaO) reflect differences in molecular confinement and interfacial interactions. This is in agreement with cross-sectional SEM images, where distinct yet well-integrated layers were observed without signs of delamination, suggesting effective interfacial adhesion.

Regarding thermal stability evaluated by TGA and summarized in Figure 6B, the thermograms reveal the thermal stability and degradation behavior of the matrices as a function of temperature. Neat PLA exhibits a single degradation step, typically associated with random chain scission, occurring at ~360 °C (Td), with a sharp mass loss indicative of its relatively homogeneous structure [47]. In contrast, PAN shows a distinct degradation profile, characterized by an initial stabilization/cyclization stage (~280–300 °C) followed by a major mass loss at higher temperatures (~390–420 °C), reflecting its more complex thermal decomposition pathway. Compared with bulk PAN materials, the electrospun fibers exhibit lower decomposition temperatures due to the enhancement of chain twisting [49]. In addition, is it possible to observe a mass loss at temperatures below 150 °C indication moisture loss and residual solvent evaporation [46].

The incorporation of CaO modifies the degradation behavior of both polymers. In PLA/CaO, a shift in the degradation temperature toward lower values is observed, suggesting that CaO may catalyze thermal degradation, likely through interactions with ester groups that promote chain scission [47]. However, the residual mass at high temperatures increases, consistent with the presence of thermally stable inorganic content. Similarly, PAN/CaO sample exhibits change in its degradation profile, including shifts in degradation temperatures and increased the range of temperatures for carbonization process, indicating that CaO influences the cyclization and carbonization processes characteristic of PAN [48].

In multilayer systems, the TGA curves display combined degradation features of both polymers, with multiple reflecting the sequential decomposition of PLA and PAN phases. The PLA/PAN/CaO system shows an earlier onset of degradation, associated with the PLA phase, followed by PAN-related degradation at higher temperatures. In contrast, PAN/PLA/CaO exhibits a slightly delayed initial mass loss, suggesting that the outer PAN layers may act as a thermal barrier, partially protecting the underlying PLA.

Importantly, the absence of abrupt or discontinuous mass loss events supports a gradual degradation process rather than layer-by-layer detachment, indicating good interfacial cohesion between layers.

### 3.6. Mechanical Characterization by Uniaxial Tensile Testing

**Figure 7:**
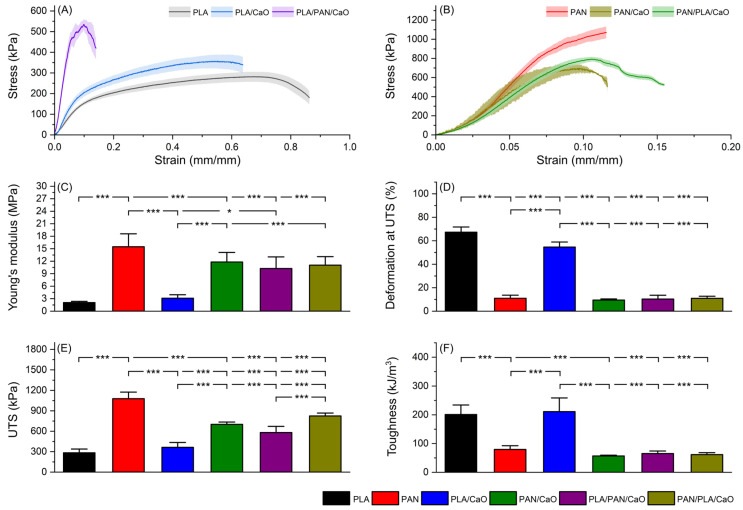
Mechanical performance of electrospun scaffolds. (**A**) Representative engineering stress–strain curves of PLA-based (PLA, PLA/CaO, and PLA/PAN/CaO), and (**B**) PAN-based mats (PAN, PAN/CaO, and PAN/PLA/CaO). (**C**) Young’s modulus. (**D**) Deformation at UTS (εUTS). (**E**) Ultimate tensile strength (UTS). (**F**) Toughness. (n = 7) (*p* < 0.05, * *p* < 0.001, ***).

Balancing stiffness/strength with conformability and structural integrity is a central requirement for electrospun wound dressings, which must tolerate handling and external stresses while remaining compliant to accommodate body motion and protect regenerating tissue [50]. Figure 7 summarizes the tensile response of the electrospun scaffolds. Representative stress–strain curves for PLA-based mats (PLA, PLA/CaO and PLA/PAN/CaO) and PAN-based mats (PAN, PAN/CaO and PAN/PLA/CaO) are shown in Figure 7A,B, while the extracted mechanical parameters are compared in Figure 7C–F. Neat PLA exhibited a highly deformable response (εUTS = 67.35 ± 4.41%) with low stiffness and strength, whereas PAN was markedly stiffer and stronger but less extensible (εUTS = 11.01 ± 2.60%). Accordingly, PAN showed a significantly higher Young’s modulus than PLA (15.48 ± 3.11 vs. 2.06 ± 0.30 MPa; ***) and a higher UTS (1079 ± 97 vs. 284 ± 55 kPa; ***). Conversely, PLA displayed greater toughness than PAN (201 ± 33 vs. 79.6 ± 12.9 kJ/m^3^; ***), highlighting complementary mechanical roles consistent with electrospun wound membrane literature and prior experimental reports on PLA-based dressings and PAN-containing barrier systems [51].

CaO produced polymer-dependent effects. In PLA, CaO did not significantly change modulus or UTS (*p* > 0.05) but reduced εUTS (54.65 ± 4.28% vs. 67.35 ± 4.41%; Bonferroni-adjusted *p* < 0.0001), while toughness remained comparable (*p* > 0.05). Reductions in elongation after introducing inorganic phases into electrospun PLA systems have been associated with restricted chain mobility and local stress concentrations linked to particle dispersion [22]. In PAN, CaO significantly decreased UTS (701.8 ± 33.1 kPa; Bonferroni-adjusted *p* < 0.0001) with no significant changes in modulus or εUTS (*p* > 0.05), consistent with reports that nanoparticle loading can impair strength in PAN-based dressings when dispersion/adhesion limits effective load transfer [31].

The multilayer CaO-containing hybrids exhibited intermediate properties, bridging the gap between compliant PLA and high-strength PAN (E ~10–11 MPa; UTS 581–825 kPa). Similar intermediate profiles have been reported for multilayer PLA–PAN electrospun matrices and bilayer wound dressings [17]. Notably, deposition sequence affected peak strength: PAN/PLA/CaO showed a significantly higher UTS than PLA/PAN/CaO (825.2 ± 42.5 vs. 580.9 ± 90.7 kPa; ***), whereas modulus, εUTS, and toughness did not differ (*p* > 0.05), indicating that layer order primarily modulates maximum stress-bearing capacity. No evident macroscopic delamination was observed in the multilayer scaffolds within the tested deformation range, suggesting that the layers remained mechanically coupled up to the onset of failure. This observation is further supported by cross-sectional SEM analysis, which revealed continuous and well-integrated interfaces without structural discontinuities, as well as by DSC results, where the absence of fully independent thermal transitions and the presence of systematic shifts in T_g_ and T_cc_ indicate interfacial interactions between PLA and PAN. Together, these findings suggest that the multilayer system behaves as an interfacially coupled structure rather than a simple stacked assembly. Overall, the data indicates three distinct mechanical regimes: a compliant/high-toughness response for PLA-based mats, a stiff/high-strength response for PAN, and intermediate behavior for the multilayer hybrids. Within the hybrid designs, deposition sequence primarily influenced peak strength (UTS), while modulus, εUTS, and toughness remained comparable between layer orders [17].

### 3.7. In Vitro Relative Bacterial Viability

**Figure 8:**
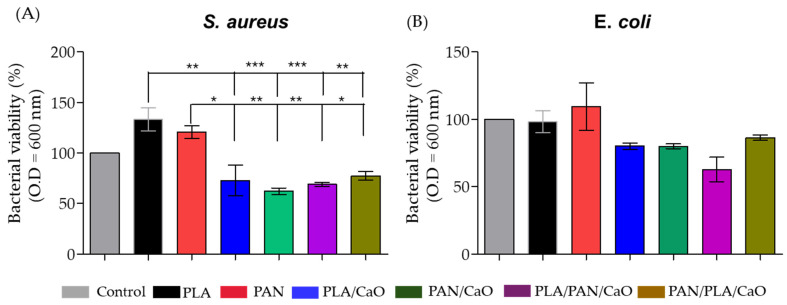
In vitro relative bacterial viability analysis of electrospun fiber using (**A**) *S. aureus*; (**B**) *E. coli* after 24 h of bacterial exposure. (n = 3); (*p* < 0.05, * *p* < 0.01, ** *p* < 0.001, ***).

The evaluation of antimicrobial activity against *E. coli* and *S. aureus* (Figure 8) revealed a clear dependence on both scaffold composition and architectural design. In the control systems (neat PLA and PAN), bacterial viability remained high, reaching 98.1% and 109.3% for *E. coli*, and 133.1% and 120.7% for *S. aureus*, respectively. These results confirm that both polymers lack intrinsic antibacterial activity and primarily function as passive structural support [30]. Notably, viability values exceeding 100%, particularly in the case of *S. aureus*, can be associated with the physicochemical characteristics of electrospun matrices. Specifically, their surface area, porosity, and fibrous topography can promote bacterial adhesion and retention, facilitate stable colonization and potentially enhance metabolic activity measured in vitro. This behavior has been widely reported for electrospun polymeric scaffolds, where surface roughness and fiber diameter play a key role in microbial attachment [52,53].

The incorporation of n-CaO induced a significant reduction in the cell viability of both microorganisms. In monolayer systems, the PLA/CaO matrix achieved a reduction of 27.2% (*S. aureus*) and 19.1% (*E. coli*), while the PAN/CaO system showed superior efficacy, with reductions of 37.8% and 20.0%, respectively. In all cases, greater susceptibility was observed in the Gram-positive strain (*S. aureus*). This selectivity is attributed to structural differences in the cell wall: the absence of an outer membrane in Gram-positive bacteria facilitates direct contact with the nanoparticles. In contrast, the outer membrane of Gram-negative bacteria acts as an additional hydrophobic barrier that limits the diffusion of cytotoxic agents [54,55]. Reports exist about similar mechanisms in PLA matrices reinforced with n-CaO, where antimicrobial efficacy is linked to the generation of reactive oxygen species (ROS) and the controlled release of Ca^2+^ ions, which induce oxidative stress and cell lysis [30]. Regarding multilayer configurations, the PLA/PAN/CaO system exhibited the greatest inhibitory potential, achieving a 31.1% reduction for *S. aureus* and a remarkable 37.1% for *E. coli*. Conversely, the PAN/PLA/CaO variant showed lower efficacy, with reductions of 22.6% and 13.7% for Gram-positive and Gram-negative strains, respectively.

The integration of n-CaO confers critical bioactive properties to electrospun scaffolds, transforming them from inert supports into active antibacterial barriers. The PLA/PAN/CaO multilayer system stands out as the most promising architecture for skin regeneration, as it optimizes nanoparticle exposure and enhances broad-spectrum bacterial inhibition. This synergy between the hierarchical structure and inorganic reinforcement is fundamental for mitigating infections during the healing process, promoting a sterile microenvironment conducive to tissue repair.

### 3.8. In Vitro Cell Viability Analysis

**Figure 9:**
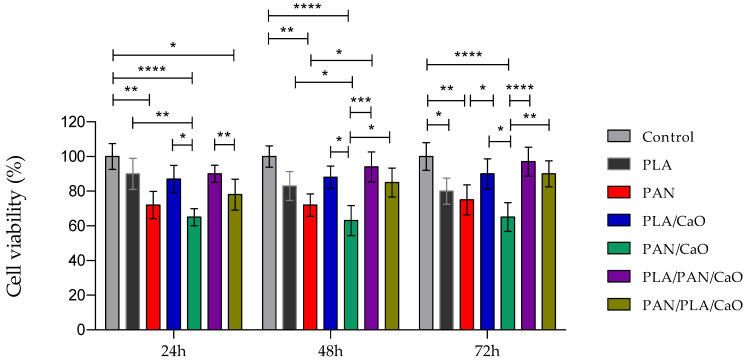
In vitro cell viability of electrospun fibers using Human Keratinocyte (HaCaT) after 24, 48, and 72 h of culture. Cell viability is expressed as percentage relative to the control. Data are presented as mean ± standard deviation (n = 3) (*p* < 0.05, * *p* < 0.01, ** *p* < 0.001, *** *p* < 0.0001, ****).

The biological behavior of the electrospun fibers was analyzed using in vitro cell viability assays with human keratinocytes of the HaCaT cell line. Culture times of 24, 48, and 72 h were evaluated, as shown in Figure 9. Overall, the PLA-based fibers performed significantly better than the PAN-based fibers (*p* < 0.05). Previous work using PLA with this cell line has shown that it is a favorable substrate for keratinocyte adhesion and proliferation [56]. For PAN matrices, previous studies indicate low viability with keratinocytes, where strategies such as functionalization with HA improve in vitro biological performance [57].

The incorporation of CaO showed a significant improvement in PLA-based matrices, with the most favorable response at 48 and 72 h. This may be related to a greater release of Ca^2+^ ions, which act as vital regulators of skin regeneration, boosting key processes such as keratinocyte differentiation, cell migration, and skin barrier repair. The ions establish a gradient that promotes epidermal renewal, trigger wound healing by stimulating fibroblast activity, and promote collagen/elastin production, thus improving skin density and hydration [25,26]. This effect was not observed in PAN-based matrices, suggesting a less favorable response.

On the other hand, the strategy of combining both matrices in multilayer systems showed that, for systems with a higher proportion of PLA (PLA/PAN/CaO) as well as those with a higher proportion of PAN (PAN/PLA/CaO), the response at all time points studied was much more favorable, indicating that structural assembly plays a key role in biological behavior. The PLA-based multilayer system performed better than the other matrices and, when compared to the PAN-based multilayer system, indicates that the outermost layer plays a dominant role in the response [17,58].

The observed keratinocyte viability profiles revealed a clear dependence on both polymer composition and scaffold architecture, supporting our initial hypothesis that a multilayer configuration with optimized surface chemistry enhances cellular compatibility. Across all time points, PLA-based membranes consistently supported higher viability than PAN-based systems, emphasizing the well-documented biocompatibility of poly (lactic acid) due to its favorable surface chemistry and degradation by-products that are generally well tolerated by keratinocytes and other skin cell types. For instance, previous studies have reported enhanced proliferation and ECM deposition on PLA nanofibrous scaffolds in skin tissue engineering applications, consistent with our observation that PLA substrates provide a more conducive platform for keratinocyte survival and growth [59,60].

Importantly, the PLA/PAN/CaO multilayer scaffold outperformed all single-layer membranes, including PLA and PLA/CaO, particularly at 48 and 72 h. This result aligns with literature showing that multilayer and composite scaffolds can synergistically combine mechanical stability with bioactive cues to promote cell viability; similar multilayer designs have been shown to enhance cell proliferation relative to single-layer constructs by modulating local microenvironments and sustained biofactor availability [17,61,62]. The enhanced performance of the PLA-dominant multilayer suggests that controlled presentation of surface chemistry and Ca^2+^ ions, potentially reducing localized alkalinity associated with excessive CaO exposure, provides a microenvironment more favorable for keratinocyte survival. In contrast, the PAN/CaO system exhibited an unfavorable early response, consistent with prior reports in which PAN’s relatively inert surface chemistry limited initial cell attachment and proliferation without extensive functional modification [25,63].

### 3.9. In Vitro Wound Closure Analysis

**Figure 10:**
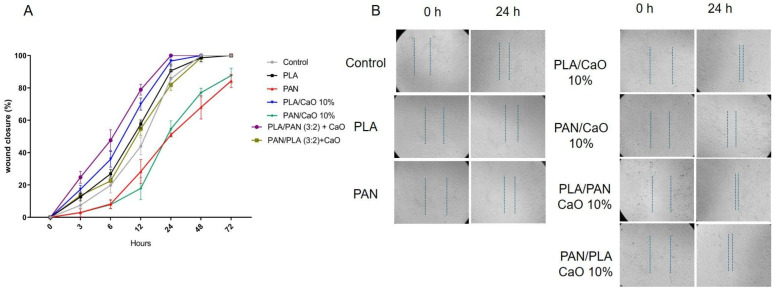
In vitro wound healing (scratch) assay of electrospun fibers. (**A**) Quantitative analysis of wound closure at 0, 3, 6, 12, 24, 48, and 72 h, expressed as percentage relative to the initial wound area. Data are presented as mean ± standard deviation (n = 3). (**B**) Representative images of wound closure in cell monolayers at 0 and 24 h for each experimental condition.

The wound-healing potential of electrospun scaffolds was evaluated using an in vitro scraping assay, monitoring wound closure for 72 h (Figure 10). All groups showed increased wound closure over time; however, marked differences in closure kinetics were observed depending on the scaffold composition and architecture.

During the early migration phase (3–12 h), PLA-containing scaffolds showed significantly faster wound closure compared to PAN-based membranes (Table 3). Notably, the PLA/PAN/CaO multilayer scaffold showed the highest percentages of wound closure at 6 and 12 h, significantly outperforming all monolayer systems (*p* < 0.05–0.001), indicating a greater migratory response of keratinocytes. Conversely, the PAN and PAN/CaO membranes showed limited wound closure during this early stage, reflecting their lower capacity to support cell migration. At 24 h, the advantage of the multilayer architecture became more apparent, with the PLA/PAN/CaO scaffold achieving almost complete wound closure, showing statistically significant differences compared to the PLA and PLA/CaO-based membranes (*p* < 0.01). The PAN/PLA/CaO multilayer also promoted wound closure more efficiently than the PAN-based systems, yet its performance remained lower than that of the PLA-dominant multilayer, highlighting the relevance of the surface-exposed polymer composition in guiding cell behavior [17]. At 48 h, complete or almost complete wound closure was observed in all PLA-based systems, while the PAN and PAN/CaO membranes still exhibited incomplete closure. At 72 h, only PAN-based membranes failed to achieve complete wound closure, confirming their limited suitability for wound healing applications [57].

Regarding the wound closure assay, scaffolds containing PLA exhibited significantly accelerated keratinocyte migration at early time points, with the PLA/PAN/CaO multilayer achieving near-complete closure by 24 h. This observation supports the notion that keratinocyte migration, a critical phase of re-epithelialization, is enhanced on scaffolds that not only support viability but also present cues favorable for cytoskeletal reorganization and adhesion dynamics. Previous work has shown that electrospun scaffolds with optimized fiber morphology and surface properties promote keratinocyte migration more effectively than less favorable substrates [12,59]. The specific advantage of the multilayer design over comparable PLA/CaO single layers highlights how architectural factors can influence early cell behavior, likely by providing differential pore structures and sustained ionic signaling that favor directional migration [6].

Overall, these results demonstrate that scaffold architecture and surface-exposed polymer composition play a decisive role in regulating keratinocyte viability and migratory behavior in vitro. The superior performance of the PLA/PAN/CaO multilayer scaffold supports the hypothesis that combining a PLA-rich biological interface with a multilayer configuration enables synergistic effects on cellular responses, surpassing those achieved by single-layer systems or alternative polymer arrangements. Importantly, the consistency between enhanced keratinocyte viability and accelerated wound closure provided a robust functional rationale for the selective progression of this scaffold to in vivo evaluation. This integrated in vitro evidence establishes a direct mechanistic link between material design and biological performance, laying the foundation for subsequent validation of tissue integration in the subdermal implantation model.

### 3.10. In Vivo Biocompatibility by Subdermal Analysis

**Figure 11:**
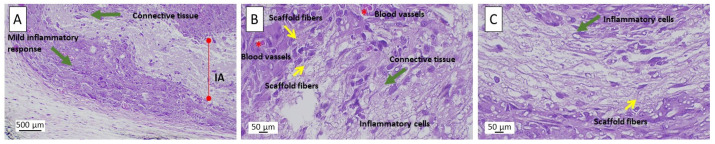
H&E-stained histological sections of subdermal tissue after implantation of the electrospun PLA/PAN/CaO multilayer scaffold. (**A**) Low-magnification overview (10×). (**B**,**C**) Higher-magnification views (40×) of the tissue–scaffold interface. Red asterisk (*) = blood vessels.

Based on functional in vitro screening, only the PLA/PAN/CaO multilayer scaffold was selected for in vivo biocompatibility assessment. Although multiple scaffold architectures were initially explored, this formulation uniquely demonstrated superior keratinocyte viability and significantly accelerated wound closure compared to all other systems. Other multilayer configurations and single-layer membranes did not achieve comparable regenerative performance. This focused evaluation strategy ensured validation of the most biologically effective scaffold while minimizing animal use in accordance with ethical research principles. Histological analysis of H&E-stained sections revealed a favorable tissue response to the electrospun PLA/PAN/CaO multilayer scaffold (Figure 11). At low magnification, the implanted scaffold was surrounded by newly formed connective tissue with no evidence of fibrotic encapsulation or necrotic regions, indicating effective tissue integration, which is commonly associated with biocompatible electrospun scaffolds [64].

At higher magnification, active cellular infiltration within and around the scaffold structure was observed. Numerous elongated fibroblast-like cells and dispersed inflammatory cells were present, suggesting a controlled inflammatory response characteristic of normal biomaterial-mediated tissue remodeling rather than chronic foreign body reaction [65]. Progressive extracellular matrix deposition and organization adjacent to the scaffold fibers were evident, consistent with connective tissue maturation as reported about regenerative biomaterial implants [66]. Importantly, newly formed blood vessels were detected in close proximity to the scaffold-surrounding tissue, indicating an angiogenic response that is critical for effective wound healing and has been widely associated with bioactive ion-releasing materials, including calcium-based systems [67]. Notably, no multinucleated giant cells or dense fibrotic encapsulation were identified, confirming the absence of adverse chronic inflammatory responses, as expected for well-tolerated polymeric scaffolds [68]. Collectively, these histological findings are consistent with previous reports demonstrating that electrospun bioactive scaffolds can promote controlled inflammation, angiogenesis, and tissue integration, supporting their application in skin regeneration and wound healing [69].

## 4. Conclusions

In this work, multifunctional hierarchical scaffolds were successfully fabricated via multilayer electrospinning of PLA and PAN reinforced with n-CaO nanoparticles. The incorporation of n-CaO induced mesoporous structures, increasing the specific surface area (up to 21.65 m^2^/g) and modulating PLA hydrophobicity (contact angle reduced from 126.3° to 91.8°). Cross-sectional SEM confirmed the presence of PLA and PAN fibers with continuous interfaces, indicating effective interfacial cohesion between them. Thermal analysis (DSC/TGA) revealed that n-CaO have an impact on chain mobility and modifies crystallization behavior, while multilayer structuring promotes interfacial interactions, supporting the formation of an integrated system rather than a simple laminate. The multilayer configurations (PLA/PAN/CaO and PAN/PLA/CaO) exhibited balanced water absorption (up to 445%), ensuring a moist environment without compromising structural integrity. Mechanically, the scaffolds displayed intermediate behavior (E ~10–11 MPa), bridging the flexibility of PLA and the strength of PAN, which is critical for mimicking skin tissue. Moreover, n-CaO conferred antibacterial activity, with PLA/PAN/CaO showing the highest inhibition against *S. aureus* (31.1%) and *E. coli* (37.1%), likely driven by ROS generation and Ca^2+^ release. In vitro assays with HaCaT cells confirmed high viability, while in vivo subdermal implantation in a murine model demonstrated good biocompatibility and tissue integration. Overall, the PLA/PAN/CaO multilayer scaffold emerges as a promising platform for skin tissue engineering, where tunable architecture enables control over mechanical, thermal, and biological performance. Future work should address long-term degradation and bioactive molecule incorporation to enhance regenerative outcomes.

## Figures and Tables

**Table 1:** Average BJH and BET values of available surface area, pore volume and pore diameter for CaO, PLA/CaO, PAN, PLA/PAN/CaO, PLA/CaO and PAN/PLA/CaO fibers.

Sample	Surface Area [m^2^/g]	Pore Volume [cm^3^/g]	Average Pore [nm]
CaO	8.96	0.02	8.04
PAN/CaO	26.93	0.12	17.29
PLA/CaO	21.65	0.05	9.16
PLA/PAN/CaO	11.43	0.09	15.35
PAN/PLA/CaO	24.16	0.05	19.19

**Table 2:** DSC thermal characterization data.

Sample	T_g_ (°C)	ΔH_g_ (J/g)	T_cc_ (°C)	ΔH_cc_ (J/g)	T_m_ (°C)	ΔH_m_ (J/g)	T_d_ (°C)	ΔH_d_ (J/g)	T_d2_ (°C)	ΔH_d2_ (J/g)	X_c_ (%)
PLA	66.6	−4.0	82.5	16.0	175.6	−44.8	362.2	−733.8	-	-	30.8
PLA/CaO	68.3	−9.6	93.3	16.4	174.8	−42.3	292.8	−303.4	-	-	30.7
PLA/PAN/CaO	68.3	−7.6	90.0	8.6	172.26	−27.1	231.1	71.3	307.2	113.3	36.6
PAN	-	-	-	-	-	-	297.3	318.9	-	-	-
PAN/CaO	-		-	-	-	-	281.7	418.6	-	-	-
PAN/PLA/CaO	67.5	−1.2	83.3	6.0	173.3	−7.0	298.3	251.3	-	-	5.9

**Table 3:** Statistically significant differences in wound closure relevant to multilayer scaffold performance.

Time	Comparison	Statistical Significance
6 h	PLA/PAN/CaO vs. PAN	*p* < 0.01
6 h	PLA/PAN/CaO vs. PLA/CaO	*p* < 0.01
12 h	PLA/PAN/CaO vs. PLA	*p* < 0.05
12 h	PLA/PAN/CaO vs. PLA/CaO	*p* < 0.01
24 h	PLA/PAN/CaO vs. PAN/PLA/CaO	*p* < 0.01
24 h	PLA/PAN/CaO vs. PAN	*p* < 0.05
48 h	PLA vs. PAN and PAN/CaO	*p* < 0.001

## Data Availability

The original contributions presented in this study are included in the article. Further inquiries can be directed to the corresponding authors.
